# Supplementary material for: Predicting Antibiotic-Associated Virulence of Pseudomonas aeruginosa Using an ex vivo Lung Biofilm Model
Source: Front Microbiol. 2020 Sep 2;11:568510. doi: 10.3389/fmicb.2020.568510 (PMC7492588; doi:10.3389/fmicb.2020.568510)
Supplement: Supplementary file 1 [file Data_Sheet_1.pdf]

Supplementary Material

Predicting antibiotic-associated virulence of *Pseudomonas aeruginosa* using an *ex-vivo* lung biofilm model

Marwa M. Hassan[*1, 2], Niamh E. Harrington[1], Esther Sweeney[1] and Freya Harrison[1]

[1]School of Life Sciences, University of Warwick, Coventry, CV4 7AL, United Kingdom

[2]Department of Pathology and Infectious Diseases, School of Veterinary Medicine, University of Surrey, Guildford, GU2 7AL, United Kingdom

*** Correspondence:**
Corresponding Author
m.hussainalihassan@surrey.ac.uk

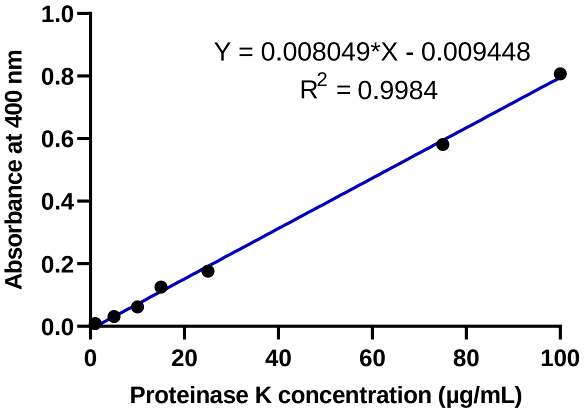

Supplementary Figure S1. Proteinase K standard curve. Proteinase K standard curve was used to calculate the concentration of total protease produced by *P. aeruginosa* using the azocasein assay.

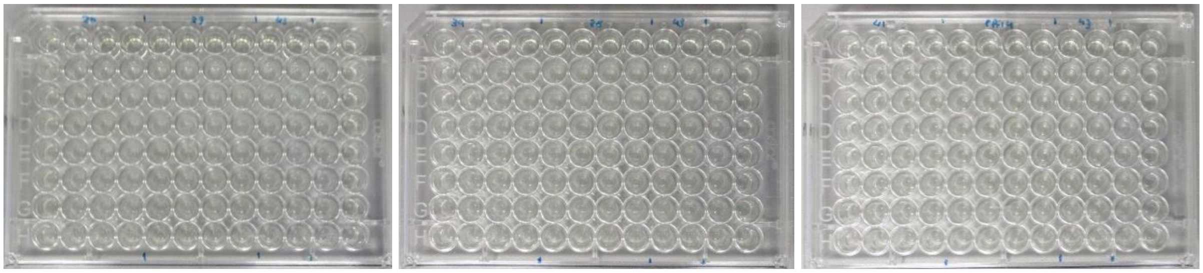

Supplementary Figure S2. MBEC recovery plates at 6 hour incubation of all tested bacterial strains. There was no visual or plate reader detection of pyochelin, pyoverdine or pyocyanin.

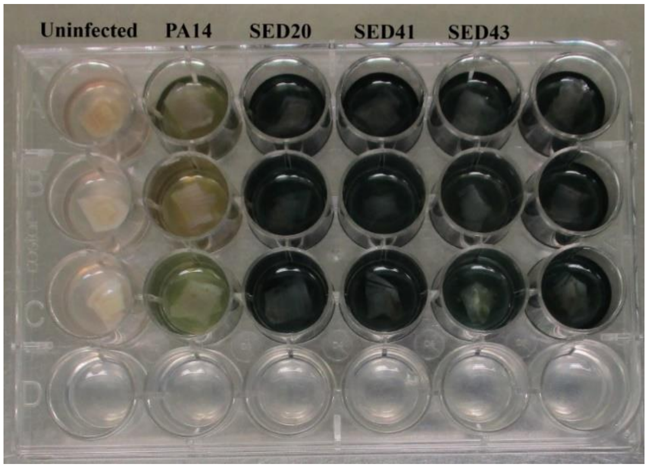

Supplementary Figure S3. Uninfected and *P. aeruginosa* infected EVPL tissues after 7 days of biofilm formation.

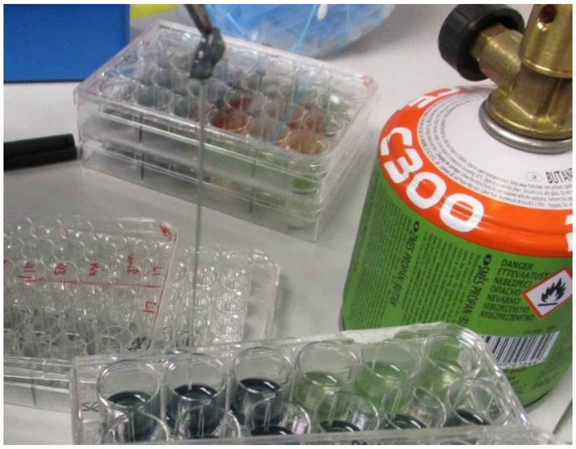

Supplementary Figure S4. Mucoid phenotype of clinical strains after biofilm formation for 7 days.

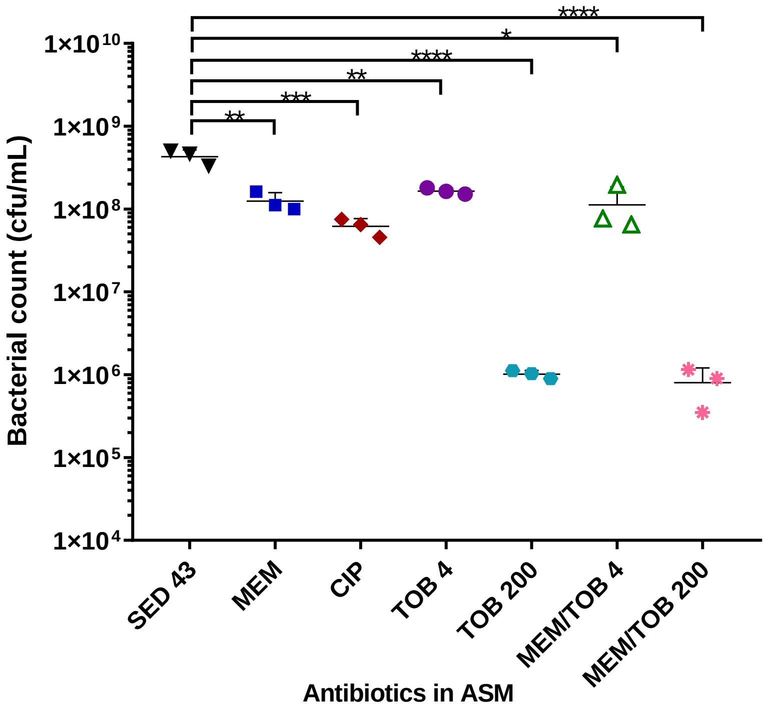

Supplementary Figure S5. Bacterial load in the EVPL with different antibiotics in ASM.

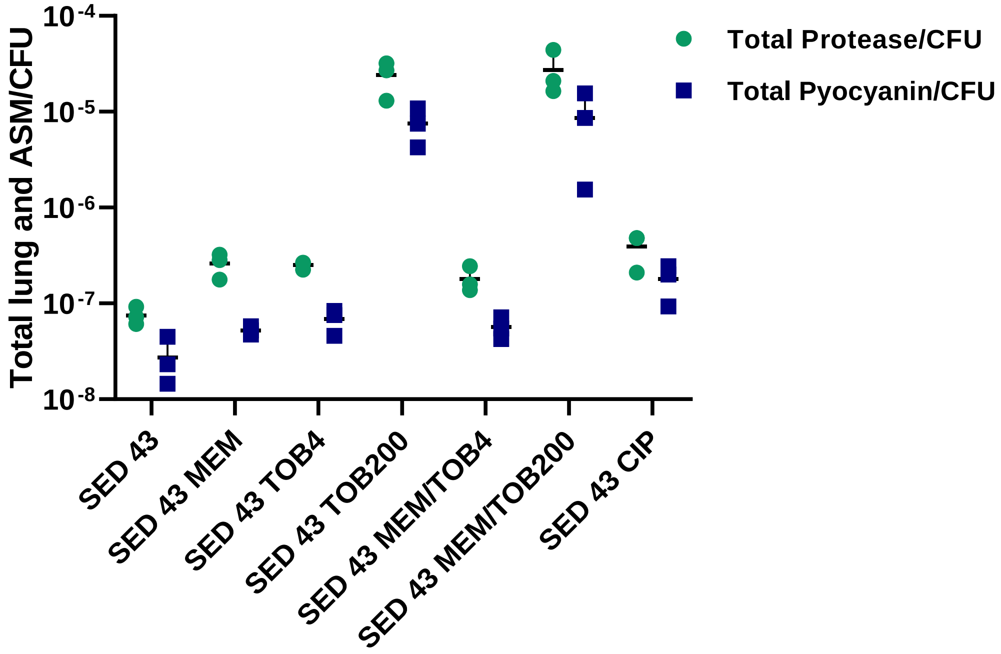

Supplementary Figure S6. Total amount of produced protease and pyocyanin/cfu in both tissue and ASM.

Supplementary Table S1. The concentration of protease and pyocyanin produced in the tissues and surrounding ASM.

Strains/conditions	Concentration of (μg/mL)			
	Protease		Pyocyanin	
	Tissue	ASM	Tissue	ASM
**Uninfected**	0.54±0.34	1.91±0.21	NA	NA
**Uninfected MEM**	1.49±0.19	2.01±0.2	NA	NA
**PA14**	6.00±0.62	63.95±4.95	2.11±0.7	8.05±3.07
**PA14 MEM**	12.12±2.73	76.22±12.02	6.85±3.48	3.94±2.96
**SED 20**	4.97±1.63	38.88±10.52	5.93±2.42	9.56±1.44
**SED 20 MEM**	7.04±4.80	50.86±18	3.45±2.62	7.02±0.75
**SED 41**	3.27±1.92	27.24±13.61	2.82±1.72	5.11±1.10
**SED 41 MEM**	5.76±3.94	43.15±24.15	3.57±3.32	9.28±7.38
**SED 43**	5.87±0.77	50.91±1.66	6.33±2.69	9.15±5.08
**SED 43 MEM**	7.22±4.89	54.85±21.47	2.81±0.71	7.67±3.38
**SED 43 TOB 4**	8.24±5.37	67.24±4.40	6.65±1.85	8.92±4.27
**SED 43 TOB 200**	3.75±1.97	39.70±12.58	4.76±1.25	5.51±4.02
**SED 43 MEM/TOB 4**	1.42±0.98	33.83±15.35	2.65±3.77	7.50±1.94
**SED 43 MEM/TOB 200**	2.20±1.17	31.07±4.40	0.77±0.34	9.29±5.74
**SED 43 CIP**	4.22±3.04	37.55±9.61	3.43±3.27	14.52±3.18

Supplementary Table S2. Fold increase in total concentration of protease and pyocyanin produced under different treatments. Blue and orange coloured values are indicating an increased and decreased percentage, respectively, in comparison to non-antibiotic treated tissues.

Conditions	Fold increase	
	Total protease	Total pyocyanin
PA14	100	100
PA14 MEM	126	106
SED 20	100	100
SED 20 MEM	132	68
SED 41	100	100
SED 41 MEM	160	162
SED 43	100	100
SED 43 MEM	109	68
SED 43 TOB4	133	101
SED 43 TOB200	77	66
SED 43 MEM/TOB4	62	66
SED 43 MEM/TOB200	59	65
SED 43 CIP	74	116
